# Using the entrapment sequence method as a standard to evaluate key steps of proteomics data analysis process

**DOI:** 10.1186/s12864-017-3491-2

**Published:** 2017-03-14

**Authors:** Xiao-dong Feng, Li-wei Li, Jian-hong Zhang, Yun-ping Zhu, Cheng Chang, Kun-xian Shu, Jie Ma

**Affiliations:** 1 Chongqing University of Posts and Telecommunications, 2 Chong Wen Road of Nan’an District, Chongqing, 400065 China; 2 Department of Bioinformatics, State Key Laboratory of Proteomics, Beijing Proteome Research Center, National Engineering Research Center for Protein Drugs, National Center for Protein Sciences (Beijing), Beijing Institute of Radiation Medicine, 38 Life Science Park Road, Beijing, 102206 China

**Keywords:** Proteomics, Tandem mass spectrometry, Entrapment sequence method, Target-decoy search, Quality control

## Abstract

**Background:**

The mass spectrometry based technical pipeline has provided a high-throughput, high-sensitivity and high-resolution platform for post-genomic biology. Varied models and algorithms are implemented by different tools to improve proteomics data analysis. The target-decoy searching strategy has become the most popular strategy to control false identification in peptide and protein identifications. While this strategy can estimate the false discovery rate (FDR) within a dataset, it cannot directly evaluate the false positive matches in target identifications.

**Results:**

As a supplement to target-decoy strategy, the entrapment sequence method was introduced to assess the key steps of mass spectrometry data analysis process, database search engines and quality control methods. Using the entrapment sequences as the standard, we evaluated five database search engines for both the origanal scores and reprocessed scores, as well as four quality control methods in term of quantity and quality aspects. Our results showed that the latest developed search engine MS-GF+ and percolator-embeded quality control method PepDistiller performed best in all tools respectively. Combined with efficient quality control methods, the search engines can improve the low sensitivity of their original scores. Moreover, based on the entrapment sequence method, we proved that filtering the identifications separately could increase the number of identified peptides while improving the confidence level.

**Conclusion:**

In this study, we have proved that the entrapment sequence method could be an useful strategy to assess the key steps of the mass spectrometry data analysis process. Its applications can be extended to all steps of the common workflow, such as the protein assembling methods and data integration methods.

**Electronic supplementary material:**

The online version of this article (doi:10.1186/s12864-017-3491-2) contains supplementary material, which is available to authorized users.

## Background

The development of mass spectrometry has provided a high-throughput, high-sensitivity and high-resolution analysis platform for proteomics. Tandem mass spectrometry has become one of the most powerful technologies for protein identification, making possible the global protein profiling. Meanwhile, using the database searching strategy allows high-throughput identification of peptides and proteins in shotgun proteomics. Varied models and algorithms are implemented by different search engines, including the early produced engines SEQUEST [1], Mascot [2] and X!Tandem [3] as well as some newly developed engines, such as Comet [4], Tide [5], MS-GF+[6] and MS Amanda [7]. Then such quality control methods have been applied to achieve high reliability identifications as PeptideProphet [8–10], PepDistiller [11], Mfs [12], RockerBox [13], FDRAnalysis [14] and BuildSummary [15].

The target-decoy database search strategy is the most commonly used strategy to estimate false identifications in target database with the assumption that the number of false identifications in target database is equal to that in decoy database [16]. However, this strategy can estimate the false discovery rate (FDR) within a dataset rather than directly evaluate the false positive matches in target identifications.

In our previous work, we used the protein sequences from *Archaea* species as appended database for standard dataset analysis to avoid the ambiguous matches caused by the sequence similarity between control protein sequences and searched database sequences [17, 18]. Similar work had been published in Granholm et al.’s [19] and Vaudel et al.’s paper [20]. Granholm et al. suggested a semi-labeled method for evaluating the calibration of a given score function using dataset of known protein sample by searching the database composed of a small number of sample sequences and a large number of entrapment sequences. Vaudel et al. proposed constructing a database that contained both the sample sequences (true positive) and entrapment sequences (false positive) and proved that the *Pyrococcu furiosus* proteome can provide a method for detecting random hits (comparable to the decoy database).

All the above-mentioned work reminds us to introduce the entrapment sequence to target-decoy search strategy as a good supplement. By using different labels, we can separate the PSMs into different kinds and calculate the false matches in target identifications directly. Using the entrapment sequence as the objective standard (pure false positive), we assessed five database search engines and four quality control methods in terms of both quantity and quality. On the basis of the results of two datasets, the entrapment sequence method is proved to be a useful strategy to assess the mass spectrometry data analysis workflow.

## Methods

### Datasets

Two previously published datasets were used in this study. The *Pfu* dataset was produced by analyzing *Pyrococcus furiosus* sample on LTQ Orbitrap Velos (Thermo Scientific) [20], and used as a standard dataset here. The *LM3* dataset was generated from a shotgun analysis of the metastatic human hepatocellular carcinoma cell line (HCCLM3) using Q-Exactive (Thermo Scientific) [21].

### Protein Sequence Database

**Fig. 1:**
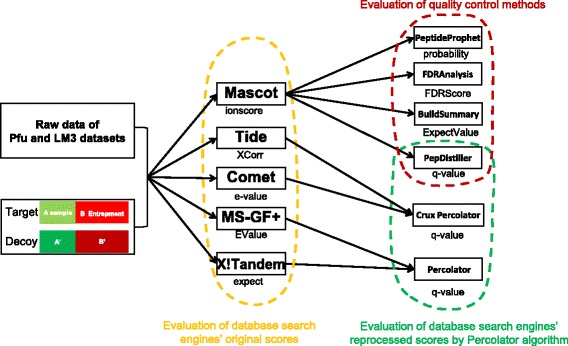
Workflow for evaluation of database search engines and quality control methods using the entrapment sequence method. A total of five search engines (Mascot, X!Tandem, Comet, MS-GF+ and Tide) and four quality control methods (PepDistiller, BuildSummary, PeptideProphet and FDRAnalysis) were studied on the basis of a standard *Pfu* dataset and a complex *LM3* dataset

Three protein sequences were downloaded from UniProt database [22]: (1) *Pyrococcus furiosus* protein sequences (*Pfu2045*, containing 2,045 sequences, downloaded on January 5, 2016). (2) *Homo sapiens* protein sequences (*Homo20187*, containing 20,187 sequences, downloaded on January 5, 2016). (3) *Archaea* protein sequences (*Arc20825*, containing 20,825 sequences, downloaded on September 21, 2016). We randomized the *Archaea* protein sequences ten times to get (4) The large entrapment sequences for *LM3* dataset (*Arc208250*, containing 208,250 sequences). The composition of the three target databases is shown in Table 1. For *Pfu* dataset, the *Pfu2045* was used as sample sequences and the *Homo20187* was used as entrapment sequences. For *LM3* dataset, the *Homo20187* was used as sample sequences, while the *Arc208250* and the *Arc20825* were used as two different entrapment sequences. Then all target sequences were reversed to create the decoy database for target-decoy search strategy. The general view of the construction of searched databases is shown in Fig. 1.

**Table 1:** Construction of the target database for *Pfu* and *LM3* datasets

DataSets	Sample sequences	Entrapment sequences	Sample tryptic peptides	Entrapment tryptic peptides	Shared tryptic peptide	Shared/Sample tryptic peptides (%)
*Pfu*	*Pfu2045*	*Homo20187*	145358	2338004	102	0.070
*LM3*	*Homo20187*	*Arc208250*	2338004	15344503	4864	0.208
*LM3*	*Homo20187*	*Arc20825*	2338004	1479773	1333	0.057

Both Granholm et al.’s [19] and Vaudel et al.’s [20] work suggested sufficiently that large entrapment sequences should be used, and that the probability that a random match hits the sample database is negligible, but the best size hasn’t been examined. Here, about ten times as many entrapment sequences were used as sample sequences, which is a similar ratio to Vaudel et al.’s work. Also, we compared the tryptic peptides of all sample sequences and entrapment sequences. As shown in Table 1, the ratios of shared peptides are respectively low for three constructed databases (0.07%, 0.21% and 0.06%). Thus, very few positive PSMs should hit the entrapment sequences. A spectrum that matches both sample and entrapment sequences is considered a sample identification.

### Database Searching

All mzML and MGF files were converted from raw files using the msconvert module [23] in the Trans-Proteomic Pipeline (TPP v4.7.0) [24]. The MS/MS peak list files were searched against the combined database using Mascot [2] (local server v2.3.2), Comet [4] (in Curx v2.1.16833 [25, 26]), Tide [5] (in Curx v2.1.16833), MS-GF+[6] (v10089) and X!Tandem [3] (TPP v4.7.0) [24]. The monoisotopic mass was used for both peptide and fragment ions with fixed modification (Carbamidomethyl, +57 Da) on Cys and variable modification (Oxidation, +16 Da) on Met. Tryptic cleavage at only Lys or Arg was selected. The miss cleavage number was set to be 1.

### Quality control and protein assembling

Four commonly used quality control methods were used in this study, including BuildSummary [15], PeptideProphet [8–10], FDRAnalysis [14] and PepDistiller [11], all of which produced a rescore of Mascot results for each PSM: BuildSummary’s ExpectValue, PeptideProphet’s probability, FDRAnalysis’s FDRScore and PepDistiller’s q-value. Comet and Tide results were processed by Percolator integrated in Crux, which gave a rescore of q-value. MS-GF+ and X!Tandem results were processed by percolator-converters (v3-00) followed by percolator (v2-08) for further quality control. The percolator tools can be downloaded from (https://github.com/percolator/percolator) [27]. In this study, we used MAYU for protein assembling [28]. Peptides less than 7 amino acids were not taken into account.

### False Discovery Rate and False Match Rate

There are two formulas commonly used for false discovery rate estimation in target-decoy search strategy. One is for seperated database search (formula (1)), and the other is for concatenated database search (formula (2)). *N*_*target*_ and *N*_*decoy*_ are the number of target and decoy matches, respectively.

1 $$ F D R=\frac{N_{decoy}}{N_{t \arg et}} $$

2 $$ F D R=\frac{2\times {N}_{decoy}}{N_{t \arg et}+{N}_{decoy}} $$

As we introduced the entrapment sequences in the target database, the entrapment hits in filtered target identifications can be considered as false positive results. Thus, we defined a false match rate (FMR) to approximately estimate the false positive identifications under given FDR. The FMR can be calculated by formula (3), where *Ntrap* is the number of identifications matched the entrapment sequences in target hits.

3 $$ F M R=\frac{N_{t rap}}{N_{t \arg et}} $$

## Results and discussion

With the advance of proteome research, a growing number of database search engines as well as the subsequent quality control methods have emerged and played the key roles in the whole process of MS/MS data analysis. As shown in Fig. 1, using the entrapment sequences as a standard, we performed the evaluation of five database search engines’ original scores and reprocessed scores and four quality control methods in the two important aspects, quantity and quality.

### Evaluation of different database search engines based on both the original scores and reprocessed scores

First, we used the *Pfu* dataset as a standard dataset to compare five search engines based on their original scores, Mascot’s ionscore, X!Tandem’s expect, Comet’s e-value, MS-GF + 's EValue and Tide’s XCorr. As shown in Additional file 1 Figure S1A-C, the MS-GF+ far outperforms the other search engines, and the use of the MS-GF + 's EValue allows significantly more identifications at all PSM, peptide and protein levels with the pre-defined FDR. The same trend has also been observed in the large *LM3* dataset (Additional file 1: Figure S1D-F).

**Fig. 2:**
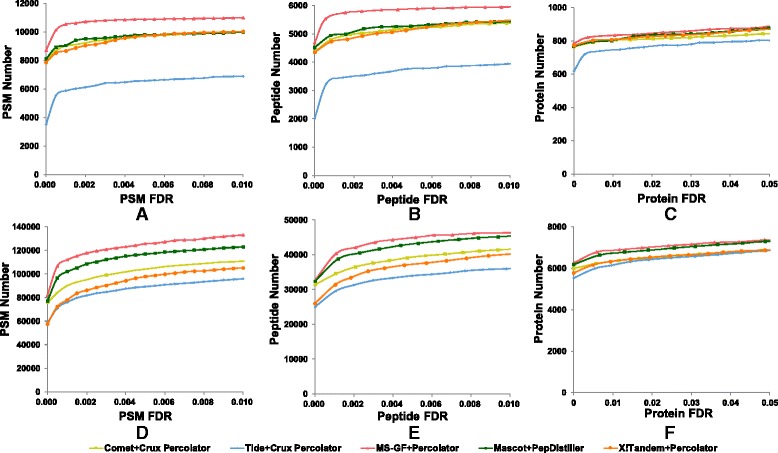
Plot figures of the numbers of PSM, peptide and protein identified by five search engines under the estimated FDRs on *Pfu* dataset (**a**-**c**) and *LM3* dataset (**d**-**f**). The reprocessed scores of all five search engines are used

The original scores of search engines are usually of very low sensitivity. However, this flaw can be overcome when combined with the followed quality control methods by considering the distributions of target and decoy hits and reanalyzing the original scores. The SVM-based percolator algorithm has been proved to be an ideal QC method [11, 29]. Thus, we further analyzed Mascot’s results by PepDistiller [11] (a bulit-in Percolator classifier), X!Tandem’s results and MS-GF+’s results by Percolator [27, 30], Tide’s results and Comet’s results by Percolator intergrated in Crux [25, 26]. All the above combinations can produce a q-value for each identification and be used for FDR calculation. As shown in Fig. 2, although the MS-GF+ combined percolator still performs best, other search engines can also perform quite well, especially in the large dataset (*LM3* dataset) and at the stringent quality control level (protein level).

**Fig. 3:**
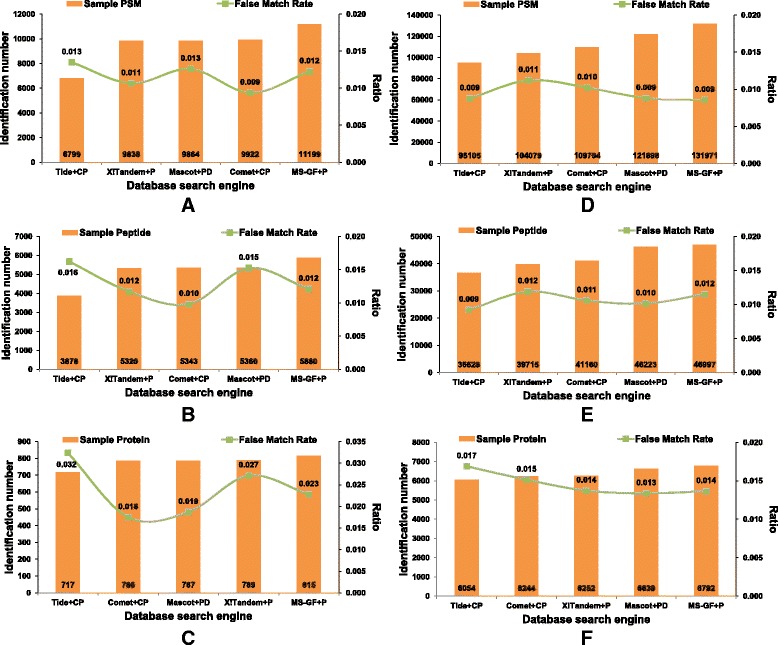
Distribution figures of the identification numbers and FMRs under 0.01 FDR of spectrum, peptide and protein level for five search engines on *Pfu* dataset (**a**-**c**) and *LM3* dataset (**d**-**f**). The reprocessed scores of all five search engines are used

In previous studies, the performance of different search engines and quality control methods were assessed by the number of results identified with fixed FDR (e.g. 1% or 5%) estimated by target-decoy hits. The most productive tool or method is usually consider the best one, which is because only quantity is used as the criterion. Here, we introduced the entrapment sequence method as a complement to the target-decoy search strategy. Thus, we can use the entrapment hits to calculate the false march rate (FMR) to assess the quality of the results. As shown in Fig. 3 (also refer to Additional file 1: Figure S2), obviously, the FDRs determined by decoy hits remain stable (0.01 FDR of PSM, peptede and protein level respectively), while the FMRs vary with search engines and confident levels.

In gerenal, fewer entrapment hits occur in PSM and peptide identificaitons and in large dataset (*LM3* dataset) than those in protein identificaitons and in small dataset (*Pfu* dataset). In most cases, the FMRs estimated by entrapment hits are roughly equal to those of FDRs estimated by decoy hits. But in some cases, the false matches represented by entrapment hits would far outnumber the expected ones, such as the Tide (FMR = 3.2%) and X!Tandem (FMR = 2.7%) searched results in *Pfu* dataset in 0.01 protein FDR condition (Fig. 3c), which would remind the researcher that more strict QC should be applied. Thus, we concluded that the entrapment sequence can be used as an internal scale for reseachers to monitor their peptide or protein identifications at any time.

### Evaluation of four quality control methods

**Fig. 4:**
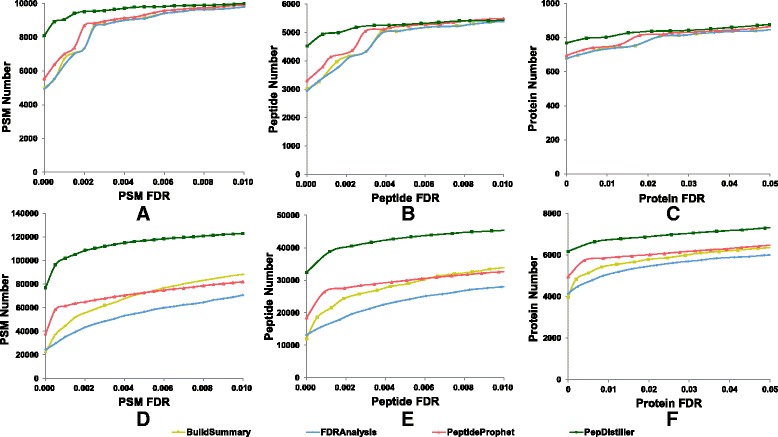
Plot figures of the numbers of PSM, peptide and protein filtered by four quality control methods under the estimated FDRs on *Pfu* dataset (**a**-**c**) and *LM3* dataset (**d**-**f**)

**Fig. 5:**
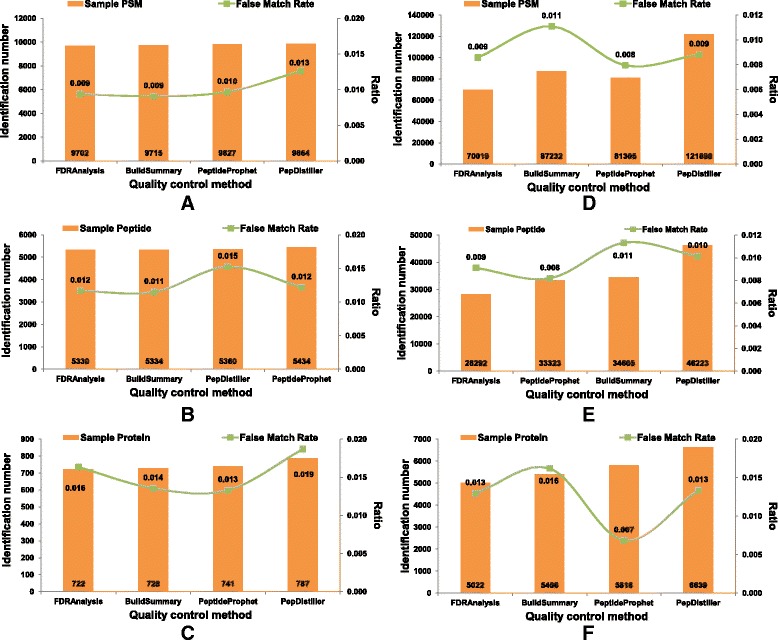
Distribution figures of the identification numbers and FMRs under 0.01 FDR of spectrum, peptide and protein level for four quality control methods on *Pfu* dataset (**a**-**c**) and *LM3* dataset (**d**-**f**)

As Mascot is one of the most widely used search engines, it has been improved to accommodate the MS/MS data generated by different instruments with different accuracy, and most quality control methods can handle its output result files. Here, Mascot’s searched files were used as inputs and reprocessed by four QC methods, including PepDistiller, BuildSummary, PeptideProphet and FDRAnalysis. As shown in Fig. 4, the percolator based QC method PepDistiller identified the most PSMs, peptides and proteins in both *Pfu* and *LM3* datasets, and other three methods were not significantly different. The trends of FMRs of filtered results by differet QC methods are close to the predefined FDR than those of search engines (Fig. 5). Using MAYU as the protein assembling tool can help four QC methods to keep confident at the peptide and protein level, especially in the large *LM3* dataset.

### Combining identificaitons of different search engines and quality control methods with an appropriate framework

Varied models and algorithms that are implemented by different search engines and quality control methods, which make themselves mutually complementary and well-performing for different subsets of mass spectrometry data. Each search engine and QC method can uniquely identify some spectra (Additional file 1: Figure S3). Indeed, combining the results of multiple database search engines or QC methods can increase identifications, however, more false positive hits will be produced by uniquely identified results.

**Fig. 6:**
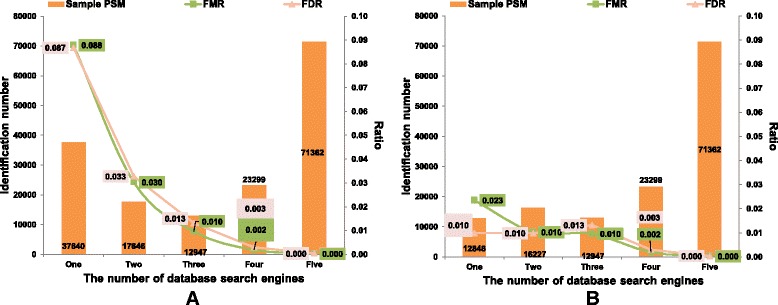
Distribution figures of the PSMs, FDRs and FMRs identified by different number of search engines on *LM3* dataset. **a** Distributions of original FDRs and FMRs under 0.01 spectrum FDR. **b** Distributions of refined FDRs and FMRs under 0.01 spectrum FDR, as the PSMs in each subgroup are further filtered to keep their sub-FDRs lower than the pre-defined one

Take *LM3* dataset as an example. Under 1% PSM FDR, the distributions of PSMs identified by one or several search engines or QC tools are shown in Fig. 6 and Additional file 1: Figure S4. Obviously, the FDRs and FMRs of peptides identified by one or two tools are much higher than those identified by three or more tools (Fig. 6a and Additional file 1: Figure S4A). If all these PSMs of five search engine are directly put together, there are 167,259 PSMs in total, resulting in 25.88% ~ 74.61% more hits than any single engine, but the FDR increases to 2.66% with the FMR of 2.61% too. Here we proposed an alternative intergrated method in which further filterings were applied to the identificaitons according to their overlap conditions. We seperated the PSMs into subgroups by the number of identified tools, and then filtered each subgroup hits to keep their sub-FDR lower than the pre-defined one (Fig. 6b and Additional file 1: Figure S4B). There are total 137,342 PSMs identified by this intergrated method, resulting in 3.37% ~ 43.38% more hits than any single engine, but the FDR decrease to 0.40% with the FMR of 0.48%.

Thus, combining the results of multiple database search engines and QC methods with an appropriate framework would benefit the data analysis process, increase the numbers of identified peptides and improve the confidence level of identifications.

### Using a small size of entrapment sequences to evaluate the search engines and tools in large dataset

As mentioned in Granholm et al.’s [19] and Vaudel et al.’s [20] papers, to efficiently separate correct PSMs from incorrect ones, the size of the entrapment sequences is supposed to be many times larger than the size of the sample sequences. However, the oversize database would greatly increase the search time while decreasing the total positive identifications. Thus, an appropriate size database is preferable in practical use. Here, we used the original *Archaea* protein sequences (*Arc20825)* as a small size entrapment sequence and reprocessed the *LM3* dataset. Then the similar results are gained as with large size entrapment sequence search (details are shown in Additional file 1: Figure S5 and S6). Thus, an easy way to use the entrapment sequence method is to randomize the sample sequences, label them and combine them with the sample sequence to construct a routine target-decoy database search, so that the entrapment hits included in each step can be used to provide a rough estimation of the confidence of the intermediate or final results.

## Conclusions

In this study, we proposed a complementary use of target-decoy search strategy for evaluation of proteomics data analysis workflow. The labeled entrapment sequences are combined with the sample sequences to construct the target database for search, then the entrapment hits can be considered as false positive results and used to access the quality of proteomics data analysis tools. Based on this method, we assessed the two key steps of the mass spectrometry data analysis process, database search engines and quality control methods. Tested by both standard and experimental datasets, we found that the new search engine MS-GF+ and the support vector machine model based quality control method PepDistiller performed best in all evaluated tools, and the performance of search engines can be improved after the combination with efficient quality control methods. We also proposed an alternative intergrated method for results from different tools. Filtering the identificaitons according to their overlap conditions, we can increase the number of identifications and improve the confidence level at the same time.

Moreover, the entrapment sequence method could be an excellent strategy to assess all steps of the mass spectrometry data analysis process. Its applications can be extended to protein assembling methods, data integration methods and so on. By objective assessment of all steps of the common MS data analysis, we can standardize the analysis pipeline of mass spectrometry data.

## Additional file

Additional file 1: This file contains supplementary figures, including **Figure S1-S6**. (PDF 477 kb)

## Abbreviations

FDR: False discovery rate
FMR: False match rate
MS: Mass spectrometry
PSM: Peptide spectrum match
QC: Quality control
