# Supplementary material for: Using the entrapment sequence method as a standard to evaluate key steps of proteomics data analysis process
Source: BMC Genomics. 2017 Mar 14;18(Suppl 2):143. doi: 10.1186/s12864-017-3491-2 (PMC5374549; doi:10.1186/s12864-017-3491-2)
Supplement: Additional file 1: — This file contains supplementary figures, including Figure S1-S6. (PDF 477 kb) [file 12864_2017_3491_MOESM1_ESM.pdf]

Using the entrapment sequences method as a standard to evaluate key steps of proteomics data analysis process

Additional file 1

Figure S1. Plot figures of the numbers of PSM, peptide and protein identified by five search engines under the estimated FDRs on *Pfu* dataset (A-C) and *LM3* dataset (D-F). The original scores of all five search engines are used.

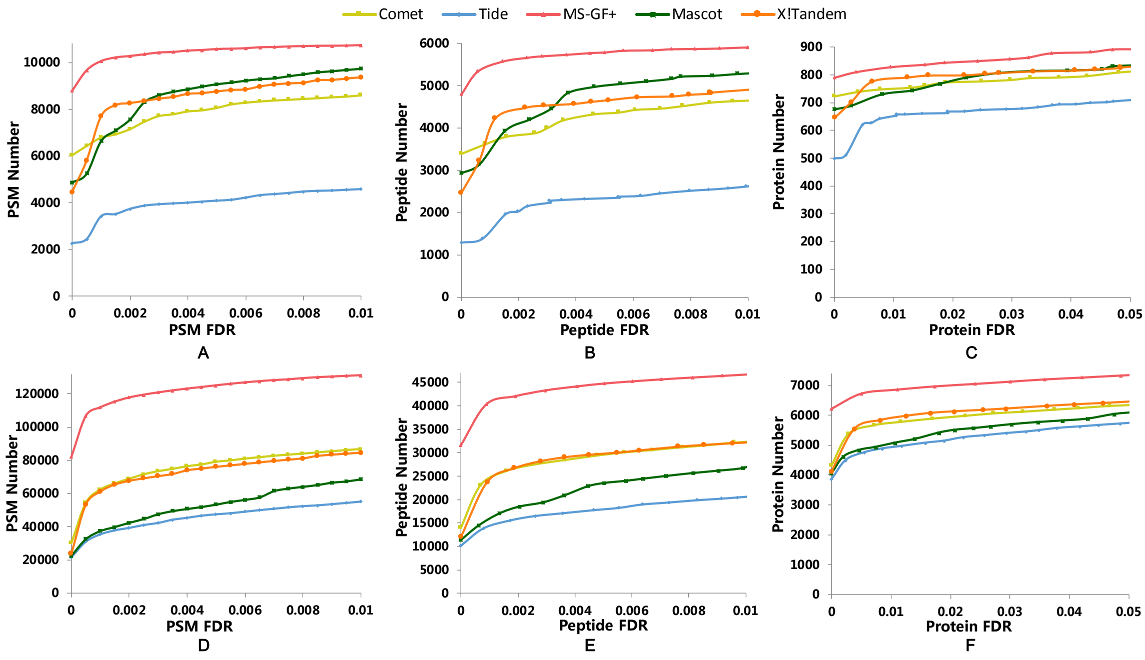

Figure S2. Distribution figures of the identification numbers and FMRs under 0.01 FDR of spectrum, peptide and protein level for five search engines on *Pfu* dataset (A-C) and *LM3* dataset (D-F). The original scores of all five search engines are used.

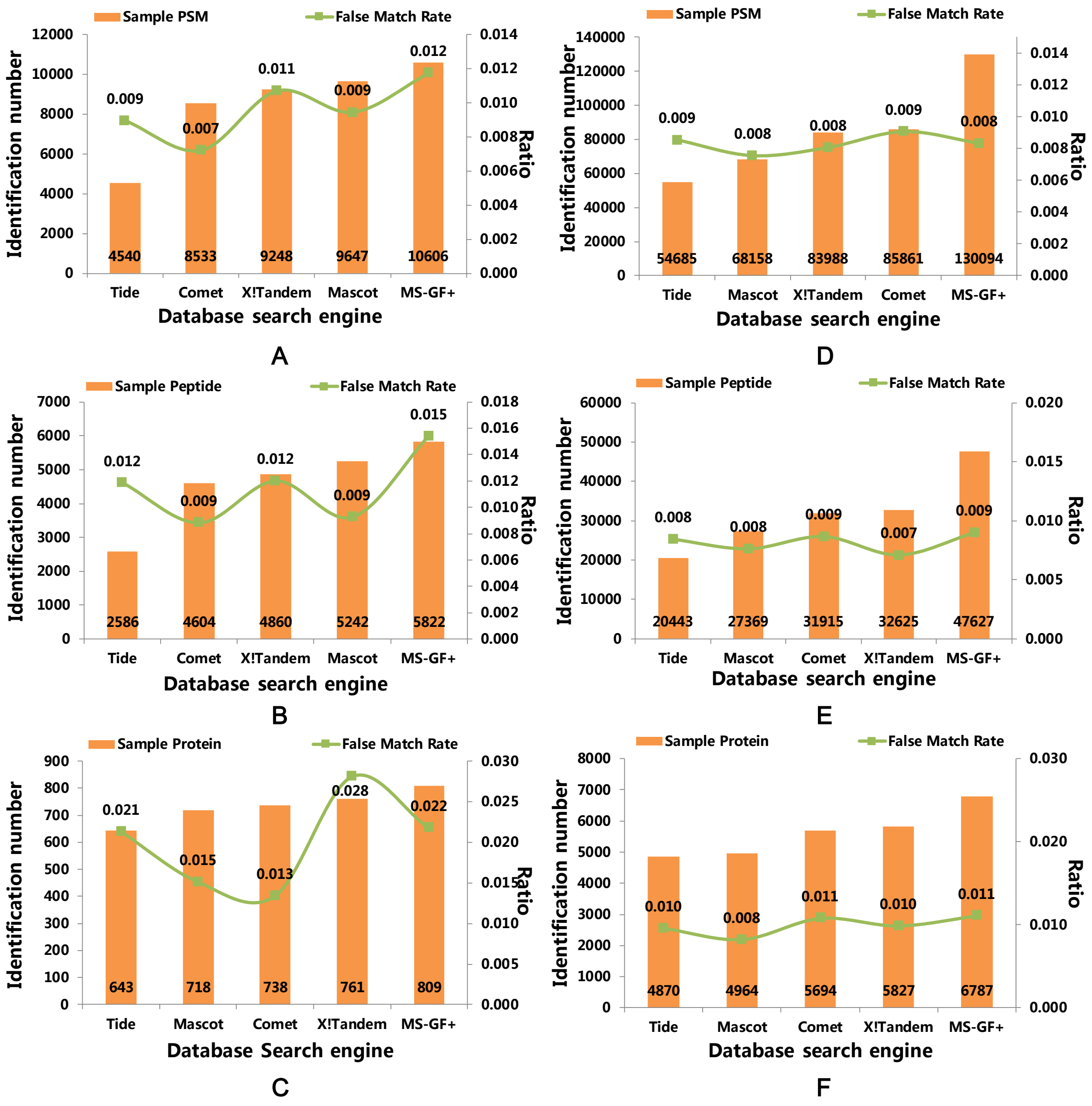

Figure S3. Overlap of PSMs identified by five search engines' reprocessed scores (A, B) and four quality control methods (C, D) on *Pfu* dataset (A, C) and *LM3* dataset (B, D) under 0.01 PSM FDR. The venn diagram was drew using the web tool on http://bioinformatics.psb.ugent.be/webtools/Venn/.

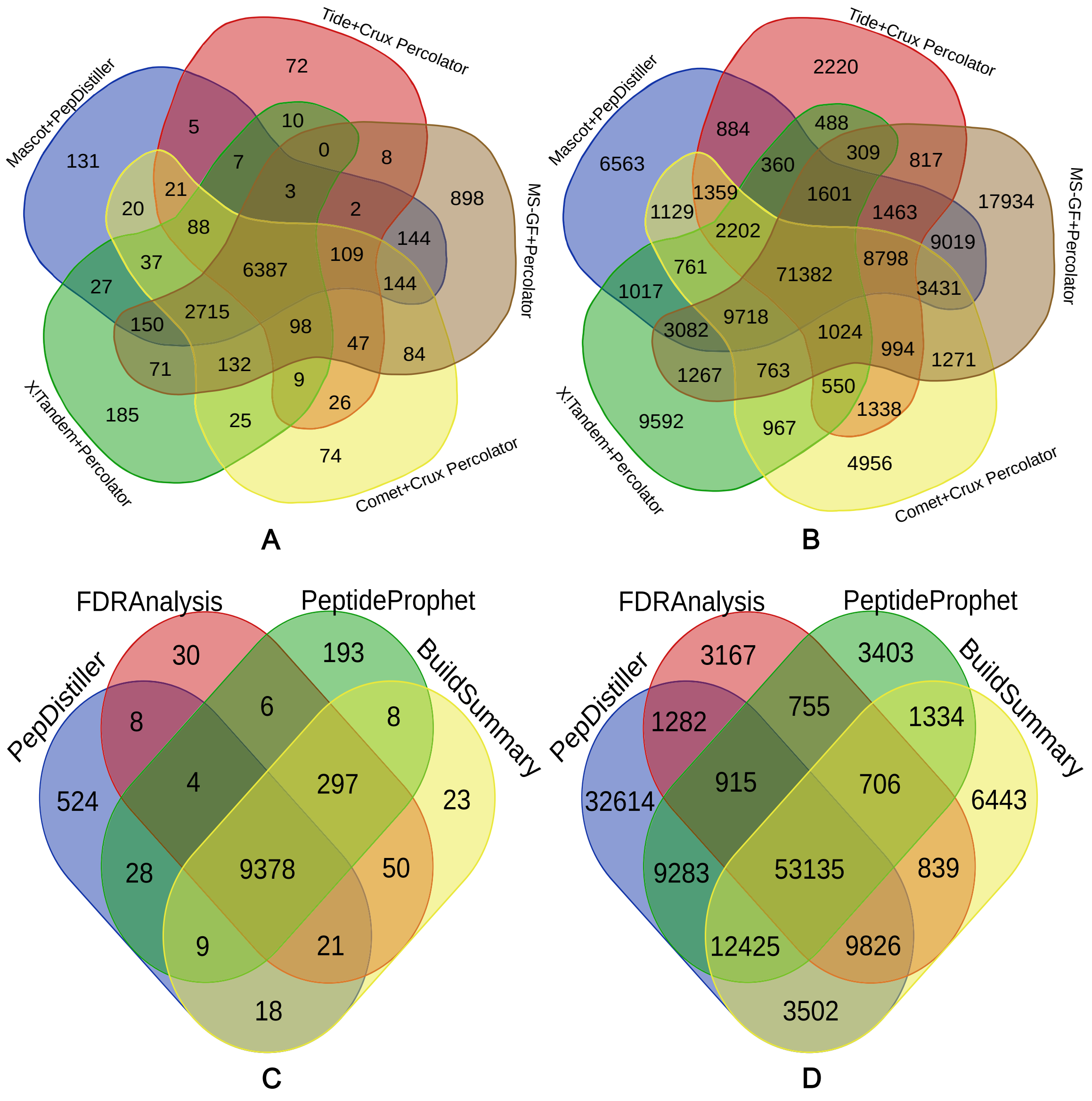

Figure S4. Distribution figures of the PSMs, FDRs and FMRs identified by different number of quality control methods on *LM3* dataset. (A) Distributions of original FDRs and FMRs under 0.01 PSM FDR. (B) Distributions of refined FDRs and FMRs under 0.01 PSM FDR, as the PSMs in each subgroup are further filtered to keep their sub-FDRs lower than 0.01 PSM FDR.

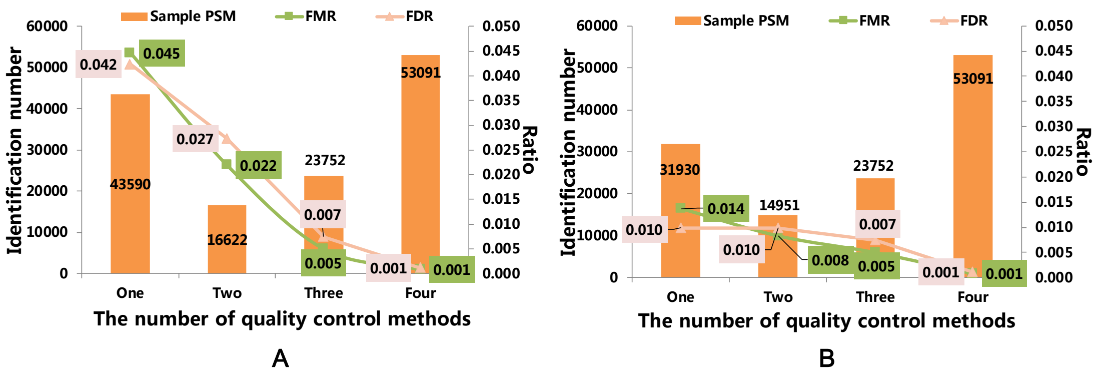

Figure S5. Using a small size of entrapment sequences to evaluate the search engines' original scores on *LM3* dataset. The original scores of all five search engines are used. (A-C) Plot figures of the numbers of PSM, peptide and protein identified by five search engines under the estimated FDRs. (D-F) Distribution figures of identification numbers and FMRs for five database search engines under 0.01 PSM FDR, peptide FDR and protein FDR.

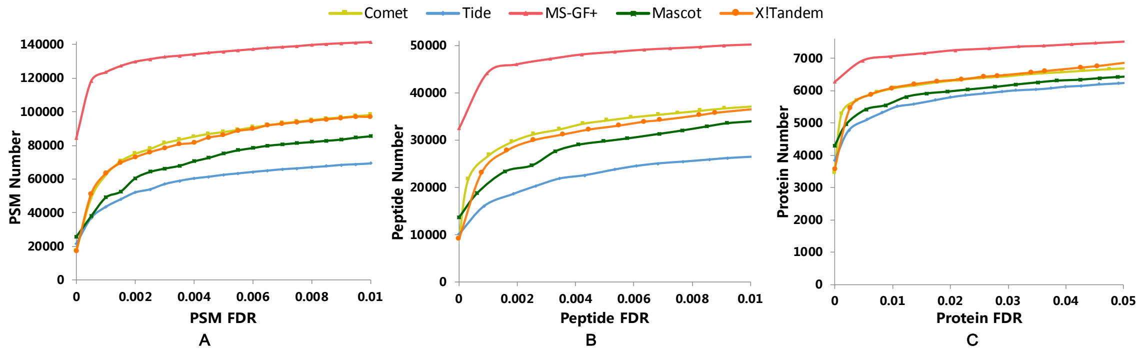

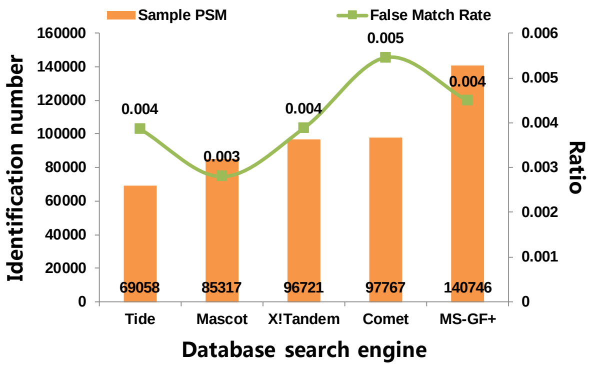
Sample PSM
False Match Rate
Identification number
Ratio
160000
140000
120000
100000
80000
60000
40000
20000
0
0.006
0.005
0.004
0.003
0.002
0.001
0
0.004
0.003
0.004
0.005
0.004
69058
85317
96721
97767
140746
Tide
Mascot
X!Tandem
Comet
MS-GF+
Database search engine

D

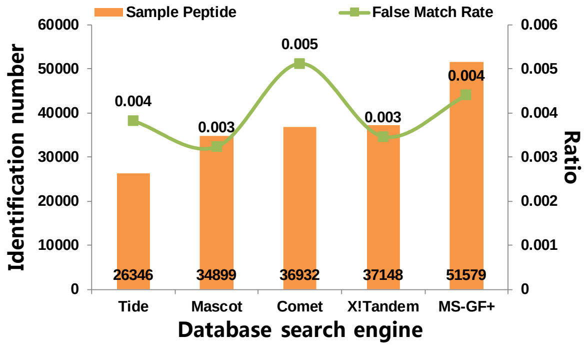
Sample Peptide
False Match Rate
Identification number
Ratio
60000
50000
40000
30000
20000
10000
0
0.006
0.005
0.004
0.003
0.002
0.001
0
0.004
0.003
0.005
0.003
0.004
26346
34899
36932
37148
51579
Tide
Mascot
Comet
X!Tandem
MS-GF+
Database search engine

E

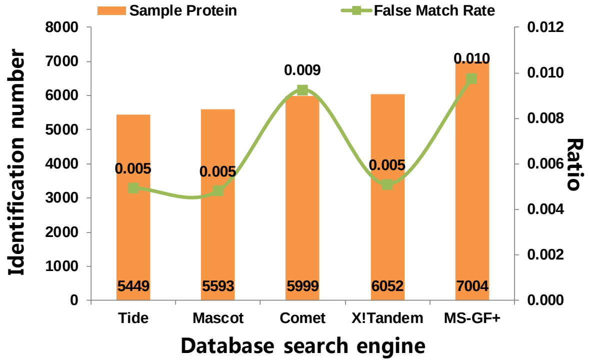
Sample Protein
False Match Rate
Identification number
Ratio
8000
7000
6000
5000
4000
3000
2000
1000
0
0.012
0.010
0.008
0.006
0.004
0.002
0.000
0.005
0.005
0.009
0.005
0.010
5449
5593
5999
6052
7004
Tide
Mascot
Comet
X!Tandem
MS-GF+
Database search engine

F

Figure S6. Using a small size of entrapment sequences to evaluate the search engines' reprocessed scores on *LM3* dataset. (A-C) Plot figures of the number of spectrum, peptide and protein identified by five search engines under the estimated FDRs. (D-F) Distribution figures of the identification numbers and FMRs for five search engines under 0.01 FDR of spectrum, peptide and protein level. The reprocessed scores of all five search engines are used. And CP, P and PD in figure stand for Crux Percolator, Percolator and PepDistiller respectively.

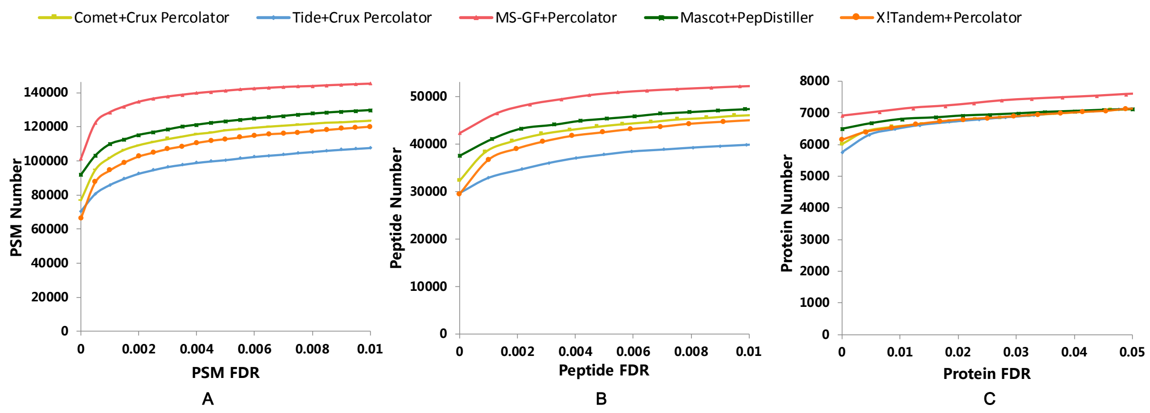

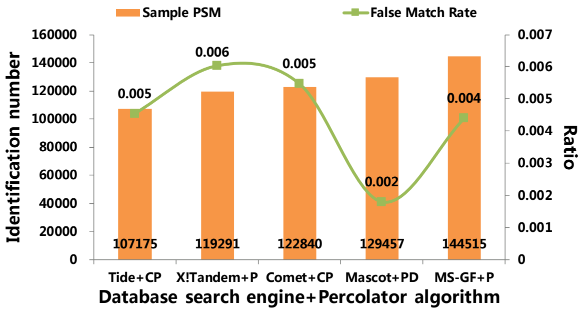
Sample PSM
False Match Rate
Identification number
Ratio
0.005
0.006
0.005
0.002
0.004
107175
119291
122840
129457
144515
Tide+CP
X!Tandem+P
Comet+CP
Mascot+PD
MS-GF+P
Database search engine+Percolator algorithm

D

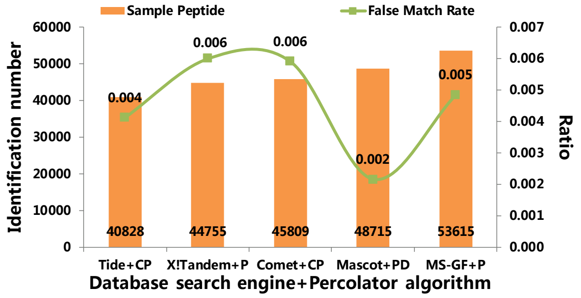
Sample Peptide
False Match Rate
Identification number
Ratio
0.004
0.006
0.006
0.002
0.005
40828
44755
45809
48715
53615
Tide+CP
X!Tandem+P
Comet+CP
Mascot+PD
MS-GF+P
Database search engine+Percolator algorithm

E

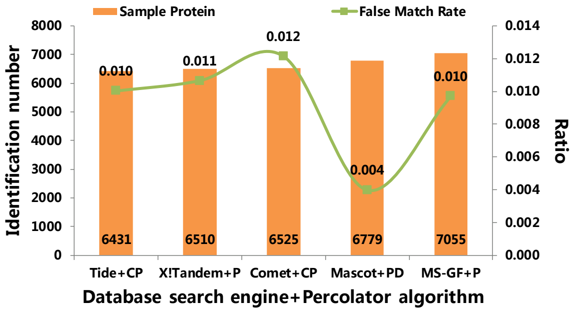
Sample Protein
False Match Rate
Identification number
Ratio
0.010
0.011
0.012
0.004
0.010
6431
6510
6525
6779
7055
Tide+CP
X!Tandem+P
Comet+CP
Mascot+PD
MS-GF+P
Database search engine+Percolator algorithm

F
